# Predictive Significance of Two MMP-9 Promoter Polymorphisms and Acetylated c-Jun Transcription Factor for Papillary Thyroid Carcinoma Advancement

**DOI:** 10.3390/diagnostics12081953

**Published:** 2022-08-12

**Authors:** Jelena Rončević, Jelena Janković Miljuš, Tijana Išić Denčić, Vesna Božić, Vladan Živaljević, Sonja Šelemetjev, Ilona Đorić

**Affiliations:** 1 Department of Endocrinology and Radioimmunology, Institute for the Application of Nuclear Energy-INEP, University of Belgrade, Banatska 31b, 11000 Belgrade, Serbia; 2 Department of Endocrine and Cardiovascular Pathology, Clinical Center of Serbia, Pasterova 2, 11000 Belgrade, Serbia; 3 Center for Endocrine Surgery, Institute of Endocrinology, Diabetes and Diseases of Metabolism, Clinical Center of Serbia, Doktora Subotića 13, 11000 Belgrade, Serbia; 4 Faculty of Medicine, University of Belgrade, Doktora Subotića starijeg 8, 11000 Belgrade, Serbia

**Keywords:** papillary thyroid carcinoma, MMP-9, promoter polymorphism, transcription factors, carcinoma progression

## Abstract

Papillary thyroid carcinoma represents a challenge from a prognostic standpoint. Molecular alterations responsible for PTC advancement include MMP-9 genetic promoter polymorphisms that bind transcription factors with varying degrees of affinity and, hence, constitute a predisposition for MMP-9 expression. We examined how two promoter polymorphisms (the -1562 C/T transition and -131 (CA)n tandem repeats) as well as levels of the c-Jun transcription factor and its modified form acetylated at Lys271 influence MMP-9 expression and PTC progression. A significant proportion of PTC samples were heterozygous for the (CA)n tandem repeat number, had a transcription-promoting T allele at -1562, and expressed high levels of c-Jun, acetylated c-Jun, and MMP-9 protein. The T allele at the -1562 position accompanied the elevated MMP-9 protein expression, while high acetylated c-Jun levels accompanied the high MMP-9 protein levels on mRNA. The -1562 C/T transition, MMP-9, and acetylated c-Jun were associated with the presence of extra-thyroid invasion and degree of tumor infiltration, while the T allele and acetylated c-Jun also correlated with tumor stage. We conclude that the -1562 MMP-9 polymorphism and levels of acetylated c-Jun affect PTC progression via modulation of MMP-9 levels. Genotyping the MMP-9 at -1562 and estimating the levels of MMP-9 and acetylated c-Jun in PTC may prove beneficial in identifying high-risk patients.

## 1. Introduction

Papillary thyroid carcinoma (PTC), the most common malignancy of the thyroid gland, represents a fairly indolent neoplasia with high survival and low recurrence rates [1]. However, due to molecular events not entirely elucidated, a relatively small proportion of patients develop a more threatening form of the disease characterized by loco-regional invasion or distant metastatic spreading. In an era of rising PTC incidence, this subset of affected patients is growing into a notable clinical challenge [2]. Despite years of research, there are currently no clinical indications or reliable molecular markers that could predict the development of unfavorable PTC phenotypes in a timely manner before the disease has progressed.

MMP-9 is a member of the matrix metalloproteinase family and a promising candidate in the pursuit of novel prognostic markers. Its primary function in extracellular matrix breakdown confers on it a protagonist role in tumor progression by removal of physical barriers, release of matrix-bound growth factors, and regulation of signaling pathways implicated in angiogenesis, migration, and proliferation [3]. Moreover, overexpression of MMP-9 has been well documented in a wide range of epithelial malignancies and is often correlated with adverse clinical features [4,5,6]. However, it is also reasonable to assume that novel candidate markers could be sought based on the reasons for MMP-9 dysregulation.

Considering that MMP-9 expression is predominately controlled at the transcriptional level [7], the inter-patient differences in MMP-9 levels could be attributed to two fundamental culprits. Firstly, inherited polymorphisms in promoter sequences of the MMP-9 gene display differential affinity for transcription factor binding and may be the root of transcription-level variations. As these are congenital traits detectable in a wide range of biological samples, they can be considered noninvasive preoperative markers denoting the initial extent of surgery. Secondly, disturbances in transcription factor levels commonly accompany the malignant process. Their expression can be detected in archival material by a pathologist to identify patients requiring more aggressive postoperative treatment.

The two most notable polymorphisms of the MMP-9 promoter are the single-nucleotide -1562 C/T transition and the copy number variation in CA repeats at the -131 position adjacent to the activator protein (AP-1) binding site [8]. The presence of the T allele at the -1562 position results in inefficient binding of a transcription-regulatory element and an increase in promoter activity [9], while the number of CA dinucleotides is speculated to affect the stability of the promoter sequence and interfere with AP-1 binding. A subunit of AP-1, c-Jun, is a part-taker in the MAPK pathway fueling the transcription of multiple genes involved in tumor invasion [10]. C-Jun undergoes substantial post-translational modifications, including phosphorylation and acetylation, which lead to changes in protein–protein and protein–DNA interactions [11]. While phosphorylation is a well-documented prerequisite for c-Jun activation, acetylation of lysine residues is still under debate. Vries et al. [12] demonstrated that c-Jun is acetylated in vivo and found Lys271 to be essential for repression of the collagenase promoter. Raivich, on the other hand, found that the acetylation of lysine residues results in c-Jun activation [11].

In the context of this conflicting evidence, in this study we attempted to re-evaluate the role of MMP-9 and a promoter sequence variation at the -1562 position on PTC progression. Furthermore, this is the first report on the distribution of (CA)n tandem repeats in the promoter of MMP-9 in PTC patients and the expression of the c-Jun transcription factor and its acetylation at Lys271.

By analyzing the expression of MMP-9 in PTC along with the molecular framework that potentially influences this expression, we tried to obtain a better understanding of the likely behavior of the tumor and, by doing so, identify molecular alterations that may serve as parallel or more valuable biomarkers in thyroid pathology. In an era of personalized medicine in which patient management decisions increasingly depend on molecular analyses, the study of the interplay between the inherited genetic background, key micro-players such as transcription factors, and effector molecules such as MMP-9 may provide vital information for tailoring the treatment to each patient’s requirements.

## 2. Material and Methods

### 2.1. Tissue Samples

A total of 50 papillary thyroid carcinoma patients who had undergone thyroidectomy at the Center for Endocrine Surgery, Clinical Center of Belgrade, Serbia were recruited for this study. Personal and clinical data on the patients, including age, gender, tumor size, presence of lymph node metastasis (LNM), degree of tumor infiltration (DI), extra-thyroid invasion (Ei), and pT status, were obtained by reviewing the pathology reports. The T value was assessed according to the 8th edition of the American Joint Committee on Cancer [13].

The degree of tumor infiltration was evaluated according to Basolo et al. [14].

Upon thyroidectomy, tumor tissue samples were divided into pieces and processed in two different ways for the purpose of establishing two separate groups of experiments. Some of the specimens from each patient were treated by formalin fixation and paraffin embedding for routine pathological examination and immunohistochemical staining. The remaining specimens were snap-frozen in liquid nitrogen and stored at −80 °C for use in DNA and RNA isolation. In parallel, a piece of conditionally healthy thyroid tissue was resected from each patient and snap-frozen as described above for use as a control sample.

All specimens were reviewed by a single pathologist, who confirmed the diagnosis of PTC according to the World Health Organization’s classification of thyroid tumors [15]. All patients were informed about the study and consented to the use of their biological material for research purposes. The study was approved by the Ethics Committee at the Center for Endocrine Surgery, Clinical Center of Belgrade, Serbia (reference no: 140/15).

### 2.2. DNA and RNA Isolation

Genomic DNA was extracted from frozen PTC samples, while total RNA was isolated both from PTC and adjacent conditionally healthy tissue from the same individual. Tissue fragments weighing approximately 0.1 g were placed on a TissueLyser (Qiagen, Hilden, Germany) with 400 µL of TRIzol reagent (Ambion, Carlsbad, CA, USA) and homogenized at 20 Hz. The isolation procedure was thereupon carried out according to the manufacturer’s instructions. The concentration of DNA and RNA was measured using a spectrophotometer and samples were preserved until further use.

### 2.3. Genotyping of the -1562 C/T MMP-9 Single-Nucleotide Polymorphism

Genotyping of the -1562 C/T MMP-9 polymorphism was performed by polymerase chain reaction—restriction fragment length polymorphism (PCR-RFLP) assay using the following primers: 5′-GCCTGGCACATAGTAGGCCC-3′ (forward) and 5′-CTTCCTAGCCAGCCGGCATC-3′ (reverse).

The PCR reaction was carried out in a total volume of 25 µL with the following cycles: initial denaturation at 95 °C for 3 min; 35 cycles at 95 °C for 30 s, 57 °C for 30 s, and 72 °C for 30 s; and final elongation at 72 °C for 7 min. The 436 bp PCR product was digested with Pael restriction enzyme (Thermo Fisher Scientific, Waltham, MA, USA) at 37 °C overnight, producing fragments of 248 bp and 188 bp in the case of a T allele and an undigested 436 bp band in the case of a C allele. Fragments were resolved on 2% agarose gel, stained with SYBR Safe (Applied Biosystems, Foster City, CA, USA), and observed under ultra-violet light. The CC genotype displayed one band with a length of 436 bp, the TT genotype gave two bands of 248 bp and 188 bp, and CT exhibited three bands (436 bp, 248 bp, and 188 bp).

### 2.4. Genotyping of (CA)n Repeats

The length of the (CA)n tandem repeat segment of the MMP-9 promoter was determined by amplifying the sequence by PCR and then resolving the fragments under denaturing conditions. The primer pairs utilized for the PCR reaction were 3′-GACTTGGCAGTGGAGACTGCGGGCA-5′ (forward) and 3′-GACCCCACCCCTCCTTGACAGGCAA-5′ (reverse).

PCR products were separated on a 12% polyacrylamide–urea gel in a formamide sample buffer and visualized after silver staining as previously described by Byun et al. [16]. As the literature states that the number of CA copies follows a bimodal distribution, the first peak being at 14 copies and the second one at more than 20 copies, in each experiment we introduced a cutoff fragment corresponding to the length of 17 repeats. By comparing the migration of each sample to the cutoff fragment, we were able to classify the alleles into a low copy number group and a high copy number group.

### 2.5. RT-QPCR

A total of 1 µg of RNA was reverse-transcribed using Revert Aid reverse transcriptase (Thermo Fisher Scientific) and random hexamer (RT) primers in a two-step running process. Quantitative real-time PCR (qRT-PCR) was performed on cDNA samples using SYBR Green PCR Master Mix (Applied biosystems), following the manufacturer’s instructions.

GAPDH was used as the reference gene to normalize mRNA levels. The reactions were performed in a 7500 Real-Time PCR system (Applied Biosystems). Each reaction was performed in triplicate and repeated twice. Inter-run controls were included in each run in order to compare the results of different experiments. The primers used for qPCR were 5′-MMP9 GCCACTACTGTGCCTTTGAGTC-3′ (forward) and 5′-CCCTCAGAGAATCGCCAGTACT-3′ (reverse) (for MMP-9) and 5′-GAAGGTGAAGGTCGGAGT-3′ (forward) and 5′-GAAGATGGTGATGGGATTTC-3′ (reverse) (for GAPDH). The relative gene expression fold change was calculated by applying a 2^–∆∆Ct^ method and all data were transformed logarithmically in order to obtain a normal distribution.

### 2.6. Immunohistochemistry

Immunohistochemical assays were performed on formalin-fixed, paraffin-embedded tissues. Briefly, the sections were deparaffinized and rehydrated. Endogenous peroxidases were blocked by hydrogen peroxide treatment for 30 min. Samples were treated with normal horse serum to prevent non-specific binding. The samples were incubated with primary and secondary antibodies. The signal was enhanced with the avidin–biotin–peroxidase complex (Vectastain ABC kit; Vector Laboratories, Newark, CA, USA) and visualized with 3,3′-diaminobenzidine-tetrahydrochloride (DAB) solution (Peroxidase Substrate Kit; Vector Laboratories, USA).

The primary antibodies used in this study were MMP-9 (mouse monoclonal, MA5-14228; Invitrogen, Carlsbad, CA, USA), c-Jun (mouse monoclonal, sc-74543, Santa Cruz Biotechnology Inc, Dallas, TX, USA), and acetylated c-Jun (rabbit polyclonal, K271, Cusabio, Houston, TX, USA).

The sensitivity and specificity of the antibodies were validated by Western blotting. Antibodies against c-Jun and acetylated c-Jun gave rise to a band at approximately 40 kDa, while MMP-9 gave a band at 92 kDa. These bands were not detected in negative control experiments when the primary antibody was omitted.

The tumor sections were examined by two individual researchers with no prior knowledge of the clinical findings. The evaluation of staining was performed in a semiquantitative manner as previously described by our research group [17]. Each slide was assigned a score as a combined measure of the intensity and distribution of the immunoreactivity in the following manner:

Score 0—no staining of tumor cells;

Score 1—weak widespread or focal (up to 40%) staining of tumor cells;

Score 2—moderate staining in more than 40% of tumor cells;

Score 3—strong diffuse staining in most tumor cells.

Cases were further grouped into a low expression group (scores 0 and 1) and a high expression group (scores 2 and 3).

### 2.7. Statistical Analysis

Statistical analysis was carried out with the SPSS 12.0.1 software package (SPSS, Chicago, IL, USA) for Windows. Student’s t-test was applied to analyze statistical differences between logarithmic gene expression fold change values. The χ^2^ test was used to check for an association between genotype frequencies, staining distributions, and clinicopathological parameters of PTC. A probability (*p*) value less than 0.05 was taken to be statistically significant. Spearman’s rank correlation analysis was employed to check for associations between analyzed parameters and the patient’s age and tumor size.

## 3. Results

Distribution of -1562 C/T and (CA)n promoter genotypes and c-Jun, acetylated c-Jun, and MMP-9 IHC scores in PTC samples

**Figure 1:**
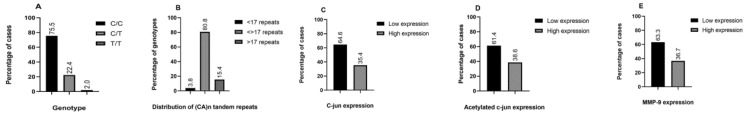
Distribution of -1562 C/T (**A**) and -131(CA)n (**B**) MMP-9 polymorphisms and c-Jun (**C**), acetylated c-Jun (**D**), and MMP-9 € expression patterns in PTC samples (**E**).

Figure 1A demonstrates that the majority of PTC cases harbored the CC genotype at the -1562 position (75.5%), while the heterozygous genotype was present in 22.4% of cases. Our sample series disclosed only one sample with the recessive TT genotype (equaling 2.0% of the samples). As described above, due to the low frequency of the rare homozygous variant, we combined it with the CT genotype for further analysis.

The distribution of CA microsatellite repeat numbers is exhibited in Figure 1B. Previously published data documented a bimodal distribution of CA copy numbers, peaking at 14 repeats and more than 21 repeats. Therefore, we introduced a cutoff value of 17 repeats and labeled the promoter alleles as either low copy number (for less than 17 repeats) or high copy number (for more than 17 repeats). After we segregated the alleles in this manner, most patients were genotyped as heterozygous (80.8%), carrying one low copy number and one high copy number of CA repeats.

The distribution of IHC staining for c-Jun, acetylated c-Jun, and MMP-9 in PTC exhibited high expression patterns in a significant number of cases (Figure 1C, Figure 1D, and Figure 1E, respectively). High expression profiles were present in 35.4% of the samples for c-Jun, 38.6% of the samples for acetylated c-Jun, and 36.7% of the samples for MMP-9. Both forms of c-Jun displayed nuclear expression; however, it should be noted that acetylated c-Jun was also present in nuclei of conditionally healthy follicles adjacent to the tumor. MMP-9 staining was membranous and sporadically detected in non-malignant elements such as endothelial cells and lymphocytes. The Spearman’s nonparametric statistical test revealed a significant correlation between MMP-9 and acetylated c-Jun expression, but not c-Jun expression (data not shown).

### 3.1. MMP-9 mRNA Expression Is Upregulated in Tumor Tissue and Correlates with Protein Expression

**Figure 2:**
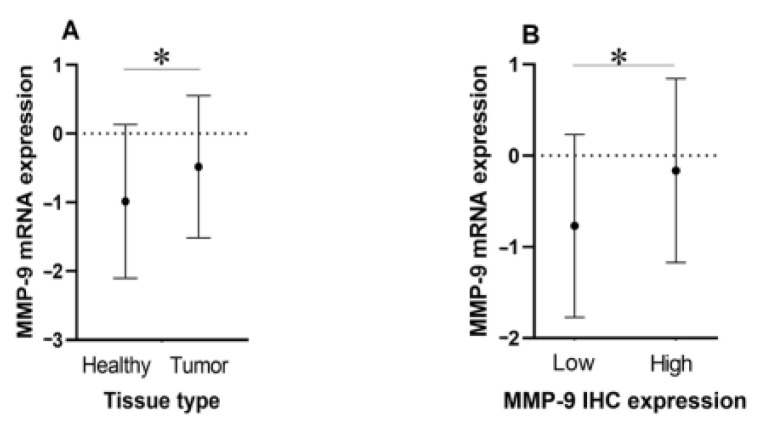
Comparison of relative MMP-9 mRNA levels between PTC and adjacent conditionally healthy tissue (**A**) and low and high MMP-9 protein expression groups in PTC (**B**). * *p* < 0.05.

Figure 2A shows the plotted mean values and standard deviations of the logarithmic MMP-9 mRNA fold change in tumor and healthy tissue. A paired-samples t-test confirmed a significantly higher mRNA expression level in malignant tissues compared with their healthy counterparts at *p* = 0.01. Figure 2B demonstrates that the patient cohort with a high protein expression level also tended to have higher mRNA levels and vice versa, again the differences being statistically significant (*p* = 0.048).

### 3.2. MMP-9 mRNA and Protein Expression in Relation to Promoter Genotype and Transcription Factor Levels

**Figure 3:**
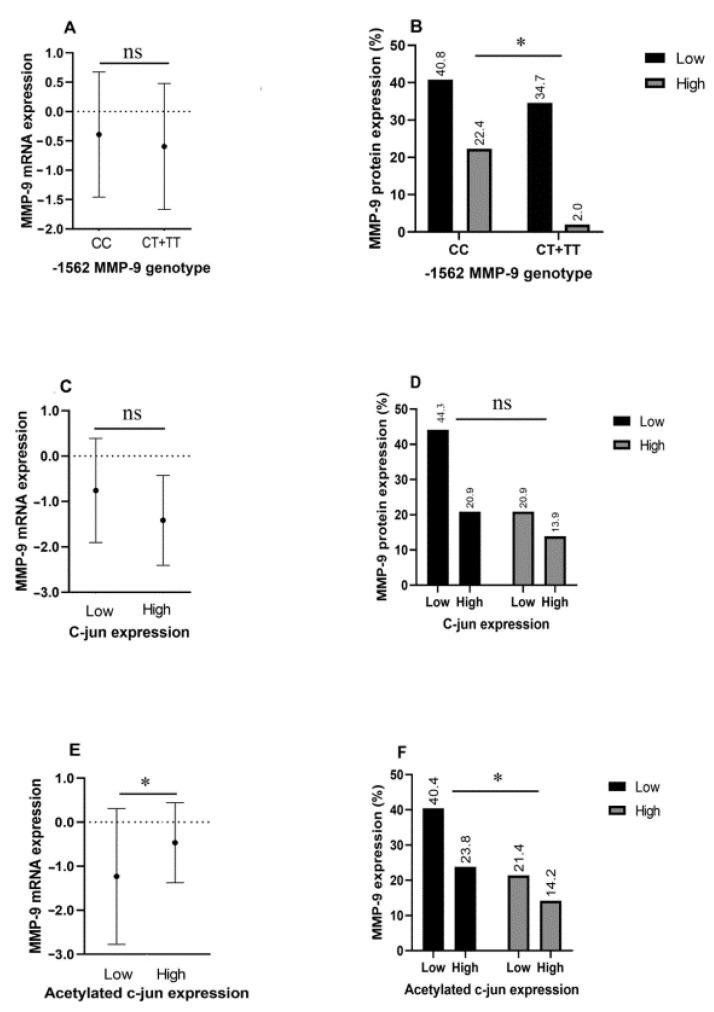
MMP-9 mRNA levels are not differentially expressed between CC genotype and T allele carriers (**A**), nor between c-Jun high and low expression groups (**C**); however, the acetylated c-Jun high expression group displayed significantly increased MMP-9 mRNA levels compared with the low expression group (**E**). MMP-9 protein expression levels differ between different genotypes at the -1562 position (**B**) and acetylated c-Jun expression groups (**F**), but not c-Jun expression levels (**D**). * *p* < 0.05.

Figure 3 illustrates how the C/T transition at position -1562 and different levels of non-acetylated and acetylated c-Jun correlate with MMP-9 mRNA and protein expression. Figure 3A illustrates differences in MMP-9 mRNA levels among CC genotype and T allele carriers; however, this inequality is not statistically significant. On the other hand, Figure 3B shows that CT carriers express significantly higher amounts of MMP-9 protein compared with their counterparts (*p* = 0.019).

As for c-Jun expression, the amount of this transcription factor did not affect either MMP-9 mRNA expression or MMP-9 protein levels (Figure 3C,D). Acetylated c-Jun levels, on the other hand, correlated significantly with MMP-9 protein and mRNA expression (*p* = 0.044) (Figure 3E,F).

### 3.3. Correlation of -1562 C/T, c-Jun, acetylated c-Jun, and MMP-9 Expression with Clinicopathological Factors of Papillary Thyroid Carcinoma

**Figure 4:**
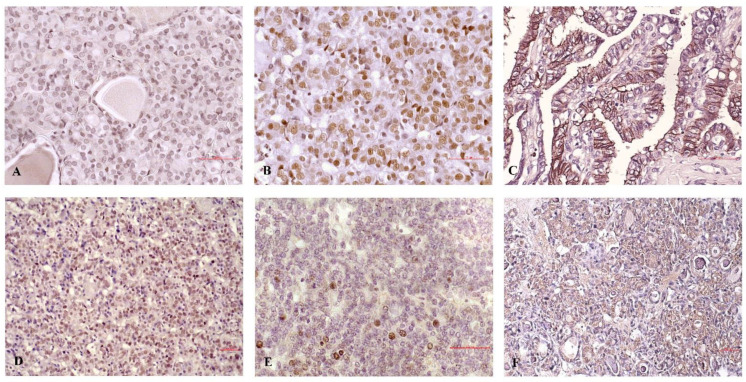
Micrograph illustrating the expression of c-Jun, acetylated c-Jun, and MMP-9 in representative PTC samples. The upper row of the micrograph represents cases with extra-thyroid invasion, showing: (**A**) weak widespread staining of c-Jun (low expression group), (**B**) strong diffuse staining of acetylated c-Jun in most tumor cells (high expression group), and (**C**) strong diffuse staining of MMP-9 in most tumor cells (high expression group). The lower row presents indolent cases of PTC, showing: (**D**) moderate staining of c-Jun in more than 40% of tumor cells (high expression group), (**E**) focal (up to 40% of tumor cells) staining of acetylated c-Jun (low expression group), and (**F**) weak widespread staining of MMP-9 (low expression group). Scale bars represent 50 µm.

The effect of -1562 C/T alteration, transcription factors, and MMP-9 levels on the development of unfavorable clinicopathological factors of PTC is summarized in Table 1. The presence of the T allele correlated significantly with the presence of extra-thyroid invasion, the T status of patients, and the depth of infiltration (*p* = 0.047, *p* = 0.002, and *p* = 0.047, respectively). C-Jun expression did not associate with any of the selected PTC features. On the contrary, levels of acetylated c-Jun had a significant effect on the development of extra-thyroid invasion and depth of infiltration (*p* = 0.029 for both comparisons). High MMP-9 expression levels corresponded to development of extra-thyroid invasion (*p* = 0.019), T status (*p* = 0.003), and depth of tumor infiltration (*p* = 0.019). Furthermore, the presence of the T allele, acetylated c-Jun expression, and MMP-9 had a strong tendency to associate with the formation of lymph node metastases, reaching borderline statistical significance. Representative micrographs illustrating the staining of c-Jun, acetylated c-Jun, and MMP-9 in PTC samples with extra-thyroid invasion and in indolent cases are presented in Figure 4.

## 4. Discussion

The signaling pathways associated with the development, progression, and metastasis of PTC have been well described. These include the MAPK, PI3K, and PTEN pathways, all of which are triggered by external stimuli and subsequently affect a multitude of downstream effector molecules [18]. Ultimately, this leads to an overexpression of transcription factors that modulate the expression of genes essential for proliferation, growth, and invasion, thus shaping the progression of the disease. MMP-9 is a protein whose dysregulation is considered to be a prerequisite for the advancement of many epithelial malignancies, including PTC [19], and its promoter carries inherited genetic polymorphisms that bind transcription factors with varying affinity. The aim of this study was to elucidate how c-Jun and its acetylated post-translational modification at Lys271 affect MMP-9 transcription and expression against the genetic background of two functionally relevant MMP-9 promoter polymorphisms, as well as their impact on PTC progression.

As a first step, we described the frequencies of the two chosen polymorphisms and levels of c-Jun, acetylated c-Jun, and MMP-9 in our PTC sample series. A smaller, but considerable proportion of our specimens were characterized by the presence of the transcription-stimulating T allele at -1562 and relatively high expression of transcription factors, while the vast majority of cases were heterozygous for the CA tandem repeat number. Interestingly, the distribution of MMP-9 staining was very similar to the staining distribution of transcription factors. We find these data to be novel and valuable as, to the best of our knowledge, this is the first description of the (CA)n repeat number in the MMP-9 promoter and c-Jun expression in PTC. Previous reports of c-Jun’s importance in the genesis of PTC come from a bioinformatic analysis conducted by Chen and collaborators in 2017 [20], which concluded that c-Jun is closely related to the onset of PTC, and a recent study by Xiao et al. [21] reporting an overexpression of AP-1 in PTC. Although the obtained distribution of CA dinucleotide repeats undermined our efforts to assess their effect on MMP-9 expression or clinicopathological factors of PTC, the aforementioned findings on -1562 C/T and c-Jun encouraged us to pursue that line of research. First, we determined that MMP-9 mRNA levels were significantly higher in malignant compared with non-malignant tissue, and, moreover, the amount of mRNA correlated with protein levels, both findings being in accordance with previously published data [22,23]. Next, we evaluated the effect of promoter polymorphism and transcription factor levels on MMP-9 mRNA and protein levels. Our data indicate that the T allele correlated significantly with elevated MMP-9 on a protein level, as we also confirmed in a previous paper [24], but not at the mRNA level, which is contrary to most reports, but could be resolved in the future by increasing the sample size. The overall pool of c-Jun protein levels did not impact upon MMP-9 expression, but only the acetylated form. Therefore, our results favor the hypothesis that acetylation of c-Jun at Lys271 increases the transcriptional activity of targeted genes. An interesting study from 2016 [25] supporting our findings reported that hyperacetylation of AP-1 increases its binding to the MMP-9 promoter and transcriptional activity.

The final focus of the paper was to analyze each of the datasets with respect to clinicopathological parameters of PTC. Many molecular–genetic studies conducted in various tumor types have revealed a frequent association of the T allele with increased levels of MMP-9 as well as with hallmarks of tumor progression [24,26,27]. A separate line of experimental studies repeatedly corroborated the relationship between MMP-9 and disease progression. Yet, sometimes there is conflicting evidence and a few exceptions can even be found. There are reports claiming a fluctuating role of MMP-9 during carcinoma progression [28], a suppressive role in colon and colorectal cancer [29,30,31], and, in some cases, no effect at all [32]. The role of c-Jun and its utility as a prognostic marker of PTC have not previously been documented; however, a recent study found a linear trend relationship between AP-1 expression and tumor size [21]. Therefore, the most important contribution of our study is the elucidation of the prognostic potential of MMP-9, its promoter variant at -1562, and c-Jun, both in the acetylated form and in total. All of the aforementioned candidates except for c-Jun proved to be promising predictors of an unfavorable clinical course of PTC. All three were excellent predictors of the development of extra-thyroid invasion and depth of tumor infiltration, while T allele and acetylated c-Jun expression also correlated with T stage. There was a strong tendency (reaching borderline significance) of the T allele, high levels of acetylated c-Jun, and MMP-9 expression to predict metastasis to lymph nodes. Hence, our data strongly suggest that the inherited genetic susceptibility portrayed by the rare allele in the MMP-9 promoter and inappropriately expressed c-Jun acetylated at Lys271 have an impact on MMP-9 overexpression, which consequently promotes PTC progression. 

## 5. Conclusions

Although our study is preliminary and has some limitations, the main ones being the limited sample size and the lack of follow-up data, we propose that genotyping of the MMP-9 promoter at -1562, being a quick, cost-effective, and accurate preoperative marker, could be beneficial for planning the initial extent of surgery. In addition, determining the levels of acetylated c-Jun and MMP-9 may be advantageous in clinical practice as ancillary postoperative markers of disease progression for tailoring treatment after surgical resection. Therefore, we strongly encourage further experimental confirmation of our findings by other researchers in order to validate our data on a larger scale and translate them as applicable molecular markers into a clinical setting.

## Figures and Tables

**Table 1:** Association of -1562 C/T polymorphism, c-Jun, acetylated c-Jun, and MMP-9 protein levels with selected clinicopathological parameters of PTC.

	-1562C/T			c-Jun			Acetylated c-Jun			MMP-9		
	CC	CT + TT	*p*	Low	High	*p*	Low	High	*p*	Low	High	*p*
Age (mean)	52.23	38.6	0.08 ^1^	45.8	54.58	0.332 ^1^	48.28	55.08	0.239 ^1^	48.1	56.4	0.172 ^1^
Tumor size (mean)	23.34	23.11	0.95 ^1^	24.2	25.25	0.745 ^1^	26.83	18.39	0.212 ^1^	20.96	29.85	0.176 ^1^
LNM 0	29	6	0.058 ^2^	24	10	0.175 ^2^	22	9	0.092 ^2^	25	10	0.060 ^2^
LNM 1	8	6		7	7		5	8		6	8	
Ei 0	27	5	**0.047 ^2^**	23	9	0.135 ^2^	20	7	**0.029 ^2^**	24	8	**0.019 ^2^**
Ei 1	10	7		8	8		7	10		7	10	
T 1/2	30	4	**0.002 ^2^**	19	11	0.815 ^2^	15	8	0.582 ^2^	26	8	**0.003 ^2^**
T 3/4	7	8		12	6		12	9		5	10	
Degree of infiltration A/B	27	5	**0.047 ^2^**	23	9	0.135 ^2^	20	6	**0.029 ^2^**	24	8	**0.019 ^2^**
Degree of infiltration C/D	10	7		8	8		7	11		7	10	

^1^ *p*-values calculated by Spearman’s test. ^2^ *p*-values calculated by χ^2^ test.

## Data Availability

The data presented in this study are available on request from the corresponding author.
